# What guides decision-making on intravenous thrombolysis in acute vestibular syndrome and suspected ischemic stroke in the posterior circulation?

**DOI:** 10.1007/s00415-020-10134-9

**Published:** 2020-08-09

**Authors:** Björn Machner, Jin Hee Choi, Alexander Neumann, Peter Trillenberg, Christoph Helmchen

**Affiliations:** 1 Department of Neurology, University Hospitals Schleswig-Holstein, Campus Lübeck, Ratzeburger Allee 160, 23538 Lübeck, Germany; 2 Department of Neuroradiology, University Hospitals Schleswig-Holstein, Campus Lübeck, Lübeck, Germany

**Keywords:** Stroke, Nystagmus, Vertigo, Perfusion imaging

## Abstract

Intravenous thrombolysis (IVT) is rarely performed in dizzy patients with acute vestibular syndrome (AVS) or acute imbalance (AIS) even if posterior circulation stroke (PCS) is suspected. Decision-making may be affected by uncertainties in discriminating central from peripheral vestibulopathy or concerns of IVT-related harm, particularly intracerebral hemorrhage (ICH), but related studies are missing. Using an in-house register of dizzy patients coming to the emergency room, we identified 29 AVS/AIS patients who presented within 4.5 h after onset, revealed clinical signs indicative of PCS (central oculomotor signs, mild focal abnormalities), and had non-contrast computed tomography (NCCT). Patients treated with IVT (*n* = 15) were compared to NoIVT patients (*n* = 14) with regard to clinical and imaging (including perfusion computed tomography, CTP) parameters, occurrence of ICH and short-term clinical outcome (NIHSS improvement; ability to walk independently). IVT and NoIVT patients did not differ in baseline characteristics, central oculomotor signs, or clinical outcome. IVT patients more often exhibited disabling vestibular symptoms (severe dizziness/vertigo, inability to stand unsupported) and focal abnormalities than NoIVT patients. There was no ICH in either group. CTP was performed in 0% of NoIVT versus 80% of IVT patients, seven of twelve revealing posterior circulation hypoperfusion. Comparison of initial hypoperfusion (CTP) and final stroke (NCCT) revealed IVT-related benefit (smaller lesion) in three of seven IVT patients. In AVS/AIS patients with suspected PCS, disabling vestibular symptoms, focal neurological deficits, and hypoperfusion on CTP seem to direct decision-making pro IVT. In our small cohort, there were no significant IVT-related clinical benefits, no IVT-related ICHs, and salvage of brain tissue in some patients.

## Introduction

Intravenous thrombolysis (IVT) with recombinant tissue plasminogen activator (rtPA, alteplase) is the recommended therapy in acute ischemic stroke with disabling symptoms within the first 4.5 h [9, 27]. This includes both anterior (ACS) and posterior (PCS) circulation strokes [7], the latter affecting the vertebrobasilar arterial territory (brainstem, cerebellum, occipital lobes) and representing about 20% of all ischemic strokes [1]. Although not specifically tested in randomized controlled trials, patients with PCS account for 5–19% of all stroke patients receiving IVT [15] and data from observational studies and registers indicate that IVT in PCS—as compared to ACS—is associated with a similar favorable outcome and mortality and even lower risk of symptomatic intracerebral hemorrhage [7, 15, 31].

However, due to the sometimes unspecific clinical presentation and normal/low scores in the established stroke assessment scales (e.g., FAST—face arm speech test, NIHSS—National Institute of Health Stroke Scale) [2, 33, 37], PCS is three times more often missed than ACS in the acute stage [37]. Furthermore, PCS patients have longer median time from symptom onset to admission and are more likely to arrive at hospital beyond the 4.5 h time window [29], which can imply increased door-to-needle times [32] or a totally missed opportunity for IVT [17, 28].

Dizziness is the most common symptom in PCS, being reported by 47% of the patients [34]. Hence, PCS patients may primarily present with an acute vestibular syndrome (AVS: vertigo, nystagmus, nausea/vomiting) or acute imbalance (AIS: dizziness, sudden unsteadiness of stance and/or gait, no nystagmus) [12, 18, 21, 36, 42]. On the other hand, only 3–4% of all patients presenting with dizziness to the emergency department (ED) really suffer from a stroke while the majority have peripheral vestibular or non-vestibular causes [13, 22]. Thus, every second PCS patient reports dizziness, but only 1 of 30 dizzy patients in the ED actually has a stroke. This may lead to an underestimation of stroke as a potential cause of acute dizziness, which is in line with the finding that this symptom is tightly linked to a missed stroke in ED patients [23, 37].

Identifying the patient with a PCS among many other dizzy patients with benign peripheral-vestibular or non-vestibular diseases in the ED may be quite easy if the patient shows severe focal neurological abnormalities (e.g., hemiparesis, aphasia/anarthria, or hemianopia) that clearly point to a lesion in the central nervous system (CNS). Furthermore, these major deficits are usually regarded as clearly disabling symptoms that per se justify the administration of IVT, irrespective of the vestibular impairment. However, there is considerable uncertainty in making the diagnosis of PCS and initiating IVT if the patient primarily presents with an AVS/AIS and no, vague or mild clinical signs of CNS involvement, e.g., focal abnormalities like hemiataxia, dysarthria and sensory deficits or central oculomotor signs [18, 28]. Even the HINTS triad (head impulse, nystagmus, test of *s*kew), which has been shown to have a high sensitivity for detecting stroke as a central cause of AVS [12], has not been investigated in the context of IVT decision-making.

Making the diagnosis of PCS in AVS/AIS patients can be supported by brain imaging studies; however, their diagnostic value, particularly of the widely used non-contrast computed tomography (NCCT), is low for detecting acute ischemic strokes in the posterior fossa [21, 28]. CT perfusion (CTP) has additional diagnostic value to NCCT for detecting acute PCS [35, 39] and CTP was previously shown to guide IVT in ACS patients in extended time windows [20]. MR perfusion imaging is less available but known to be helpful in early detecting PCS [4, 5]. Furthermore, a (cerebellar) hypoperfusion on MR perfusion imaging was previously shown to support decision-making on IVT and to illustrate IVT-related salvage of brain tissue by comparison to the final stroke lesion [16].

Taken together, the acute management of AVS/AIS patients with suspected PCS is challenging [6, 28, 36]. Decision-making of IVT in these patients requires rapid distinction between a central and peripheral cause of the dizziness syndrome and an evaluation whether the expected benefit outweighs the potential harm of IVT in the individual patient. This represents a time-critical process, because earlier IVT treatment is associated with better functional outcome [9].

Given the lack of prospective clinical trials, we used a single-center register [21] to retrospectively evaluate the diagnostic management and acute treatment (IVT) as well as the short-term outcome of AVS/AIS patients with suspected PCS presenting to the ED. We were especially interested in clinical and/or imaging parameters that may have guided decision-making in favor of IVT in this subgroup of acute dizzy patients.

## Materials and methods

### Study design and setting

This retrospective study is based on data from an in-house register of dizzy patients that was compiled by reviewing medical records of adult patients who presented with dizziness, vertigo or imbalance to the emergency department (ED) of our University Medical Center (Lübeck, Germany) between January 1, 2016 and December 31, 2018 and who received neurological work-up after exclusion of general medical causes [21]. The 610 cases in the original database were amended by 4 more patients presenting with acute dizziness to our hospital in 2019 who were identified via search for the procedure code of ‘IVT’ in the hospital’s medical controlling database.

The study was approved by the Ethics Committee of the University of Lübeck (18-146A).

### Study population

Within the register described above, we searched for patients who met the following eligibility criteria: (i) new and persistent vertigo with spontaneous nystagmus (AVS) or unsteadiness of stance and/or gait without spontaneous nystagmus (AIS), (ii) an interval of < 4.5 h between symptom onset and time of presentation at the ED, (iii) a NCCT performed in the ED and (iv) admission to the stroke unit because of suspected PCS. We excluded patients with (i) any major neurological deficit (including hemiparesis, hemianopia, and aphasia) on the neurological examination in the ED, (ii) normal stance and gait, (iii) intracranial bleeding on NCCT, and (iv) pre-existing anticoagulation with vitamin-K or non-vitamin-K antagonists.

Based on the acute treatment the patients received, those with IVT were assigned to the IVT group while the others constituted the NoIVT group.

### Clinical parameters at baseline

We collected information on demographics (age, sex), vascular risk factors, and comorbidities (arterial hypertension, diabetes mellitus) as well as the past medical history (prior stroke/TIA, any vestibular disorder including Meniere’s disease, vestibular migraine, vestibular neuritis, BPPV). We collected information on the presence of the following vestibular symptoms that may be regarded as disabling symptoms: (I) perception of very severe dizziness/vertigo reported at admission, (II) presence of nausea and vomiting and (III) the inability to stand unsupported during examination in the ED. From the patients’ neurological examination documented in the ED, we further extracted information on focal abnormalities, i.e., dysarthria, unilateral facial weakness, limb ataxia, or sensory impairments. Notably, major deficits such as aphasia/anarthria, hemiparesis, or hemianopia were exclusion criteria in this study and, therefore, not present in this cohort. We obtained the National Institute of Health Stroke Scale (NIHSS) score for each patient at admission. Furthermore, we collected detailed information from the oculomotor examination. This included the documented results from the HINTS examination [head impulse test (HIT), spontaneous nystagmus and test of skew], which were indicative of a central cause of dizziness if the HIT was documented as normal, the spontaneous nystagmus’ fast phase alternated with gaze (or bilateral gaze-evoked nystagmus), or a skew deviation was observed with or without the cover test. Other relevant oculomotor (OM) signs included a central pattern of the spontaneous nystagmus (vertical or purely torsional), eye muscle or gaze palsies (including internuclear opthalmoplegia, INO), unilateral ptosis or Horner’s sign.

### Imaging parameters

Brain imaging studies conducted at admission always included NCCT, sometimes amended by CT angiography (CTA) and/or CTP. Follow-up imaging encompassed NCCT and/or MRI.

The results of the brain imaging studies were abstracted from the official neuroradiological reports and type and localization of stroke as well as any abnormalities on perfusion imaging were double-checked by an experienced board-certified neuroradiologist among the authors (A.N.).

The CT examinations were acquired on 64 or 128 dual slice CT scanners (Somatom Definition AS or Somatom Definition AS + ; Siemens Healthcare, Forchheim, Germany). CTP post-processing was performed on a designated workstation (Syngo. Via; Siemens Healthcare, Forchheim, Germany). A deconvolution model using a least squares fitting process furnished calculation of quantitative perfusion parameter maps for cerebral blood volume (CBV), cerebral blood flow (CBF) and time to drain (TTD). A local hypoperfusion was defined as an area of prolonged TTD with or without correspondingly reduced CBF. We chose TTD as the main marker since TTD was shown to be very sensitive to all kinds of hemodynamic disturbances (i.e., normal TTD predicts regular perfusion with high probability), to well assess the extent of an ischemic lesion with high image quality and to show an excellent interrater agreement [38].

For IVT-treated patients, who initially received CTP imaging revealing focal hypoperfusion in the posterior circulation, we related the area of the initial hypoperfusion to the presence/size of the final stroke lesion on follow-up NCCT to assess any discrepancy, particularly whether the area of initial hypoperfusion was larger than the final stroke lesion, which could be taken as an indicator of IVT-related salvage of brain tissue. We also compared the individual CBF and CBV maps, to look for a potential CBF–CBV mismatch on initial CTP imaging as it is used to identify ischemic but not yet infarcted brain tissue in stroke patients with unclear or extended time windows > 4.5 h to still initiate IVT treatment [20].

The MRI scans always included axial T2 fluid-attenuated inversion recovery (FLAIR) and diffusion-weighted imaging (DWI) sequences, which were obtained on a 1.5 or 3.0 T MRI scanner (Achieva or Ingenia; Philips Healthcare, Amsterdam, The Netherlands).

### Clinical outcome measures

We evaluated the final clinical diagnosis the patient received at discharge from the hospital, particularly whether it was a PCS or a non-stroke diagnosis, e.g., vestibular neuritis.

We searched for IVT-related symptomatic intracerebral hemorrhage as defined by the SITS-MOST criteria (parenchymal hemorrhage type II and neurological deterioration with at least 4 points on the NIHSS) [40].

Clinical outcome parameters comprised the NIHSS score at discharge and its improvement with regard to the NIHSS score at admission. Furthermore, we assessed whether patients could be discharged home, able to walk independently, or whether they needed further in-patient rehabilitation.

### Statistical analysis

Statistical analyses were performed using SPSS 22.0 (IBM Corp., Somer/NY, USA). Descriptive statistics were calculated for all variables of interest, data are presented as counts and percentages. Differences between the groups (IVT, NoIVT) were statistically compared using *t* tests for quantitative variables (e.g., age) or the Pearson’s Chi-square test for categorical variables. The significance level was set at *p* < 0.05.

## Results

### Number of eligible AVS/AIS patients and distribution to IVT and NoIVT

From *n* = 610 patients in the original register from the years 2016 to 2018, *n* = 25 (4.1%) fulfilled the predefined eligibility criteria, i.e., they presented with AVS or AIS within 4.5 h after symptom onset, revealed minor signs of CNS involvement but no major neurological deficits, received NCCT that ruled out ICH, had no anticoagulation as pre-existing medication and were admitted to the stroke unit due to suspected PCS. Eleven patients (1.8%) were treated with intravenous rtPA (alteplase). Together with the additional *n* = 4 IVT-treated patients from the year 2019, *n* = 15 patients finally constituted the IVT group. The other patients meeting the eligibility criteria, but who did not receive IVT, were assigned to the NoIVT group (*n* = 14).

### Clinical and imaging findings at baseline and differences between IVT and NoIVT patients

Table 1 provides the demographic and clinical characteristics at baseline (as well as the clinical and imaging outcome parameters) for each patient in the IVT group, while Table 2 summarizes the individual results for the patients of the NoIVT group.

**Table 1:** Individual clinical characteristics and imaging findings of patients who were treated with intravenous thrombolysis (IVT group)

Subject-ID	Age	Sex	Time since onset (h:min)	Severe dizziness perception	Nausea and vomiting	Inability to stand unsupported	Dizziness syndrome	Central oculomotor signs	Focal neurological abnormalities	NIHSS at admission	Non-contrast CT	CT angiography	CT perfusion	Follow-up imaging	Final diagnosis	NIHSS at discharge	Discharged home
IVT-01	89	m	02:04	+	+	+	AIS	HIT normal	Dysarthria, dysphagia	1	Normal	Normal	−	CT: normal	Brainstem stroke	1	Yes
IVT-02	80	m	02:37	−	−	−	AVS	Horner right	Dysarthria, hemiataxia	3	Normal	Right VA occluded (V3)	Hypoperfusion right PICA	CT: stroke right PICA	Medulla + cerebellar stroke (right PICA)	3	No
IVT-03	67	m	01:20	−	−	−	AIS	Disrupt. smooth pursuit	Hemiataxia	1	Normal	Normal	−	MR: normal	Transient vestibular episode (remission < 1.5 h)	0	Yes
IVT-04	49	m	01:29	+	−	−	AVS	Skew deviation, vertical gaze palsy, bil. GEN	−	0	Normal	Normal	Normal	MR: stroke right thalamus	Thalamic stroke	0	Yes
IVT-05	64	f	01:08	+	−	+	AVS	HIT normal, Strabismus divergens	Hemiataxia	1	Normal	Normal	Normal	MR: normal	Vestibular neuritis (abnormal vHIT)	0	Yes
IVT-06	65	f	01:07	+	+	+	AVS	Upbeat nystagmus, HIT normal	Dysarthria	1	Normal	Right VA hypopl., left VA occluded, BA normal	VBS territory hypoperfused	CT: normal	Brainstem stroke	1	Yes
IVT-07	61	f	01:23	+	−	−	AIS	None reported	Hemiataxia	2	Normal	Right SCA occluded	Hypoperfusion right SCA territory	CT: stroke right SCA	Cerebellar stroke (right SCA)	2	Yes
IVT-08	69	m	03:23	−	−	+	AVS	Horner right	Dysarthria, hemiataxia, hemihypaesthesia	4	Normal	Right VA occluded	Hypoperfusion in right cerebellum	CT: normal	Stroke of the dorsolateral medulla oblongata	1	Yes
IVT-09	71	f	01:30	+	−	+	AVS	HIT normal, Horner right	Hemiataxia, hemihypaesthesia	2	Normal	Normal	Normal	CT: normal	Stroke of the dorsolateral medulla oblongata	1	Yes
IVT-10	39	m	01:23	+	−	−	AIS	None reported	Dysarthria, hemiataxia	3	Normal	Normal	Normal	MR: stroke of thalamus/capsula	Thalamic stroke	0	Yes
IVT-11	67	m	02:11	+	+	−	AIS	None reported	Dysarthria, hemiataxia	3	Normal	Moderate stenosis of VA4 bilateral	Normal	MR: normal	Brainstem stroke	2	Yes
IVT-12	65	m	04:03	+	+	+	AVS	Bilateral GEN	Dysarthria, hemiataxia, diss. hemihyp-/thermhypaesthesia	3	Normal	Occlusion of left VA(V2)	Hypoperfusion in left PICA territory	CT: normal	Stroke of the left dorsolateral medulla oblongata	Death, cardiac arrest	No
IVT-13	76	m	00:59	+	−	+	AVS	Skew deviation	Dysphagia, hypophonia, tongue deviation left	1	Normal	Occlusion of VA left (V4)	Hypoperfusion in left PICA and left pons	CT: stroke left PICA, slight hemorrhagic transformation	Left cerebellar and pontine stroke	1	No
IVT-14	66	f	00:53	+	+	+	AVS	Upbeat Nystagmus	Dysarthria	1	Normal	Normal	Hypoperfusion in right SCA territory, mesencephalon and thalamus	CT: stroke right SCA	Cerebellar stroke (right SCA)	0	Yes
IVT_15	41	m	01:27	+	−	+	AVS	Skew deviation, INO left, upbeat nystagmus	Dysarthria, hemihypaesthesia, hemiataxia	3	Ischemia SCA left	Normal	−	MRI: stroke bilat. SCA, left PICA, thalamus, mesenceph. and PCA	Stroke in cerebellum, Thalamus and Mesencephalon	3	No

**Table 2:** Individual clinical characteristics and imaging findings of patients who did not receive intravenous thrombolysis (NoIVT group)

Subject-ID	Age	Sex	Time since onset (h:min)	Severe dizziness perception	Nausea and vomiting	Inability to stand unsupported	Dizziness syndrome	Central oculomotor signs	Focal neurological abnormalities	NIHSS at admission	Non-contrast CT	CT angiography	CT perfusion	Follow-up imaging	Final diagnosis	NIHSS at discharge	Discharged home
NoIVT-01	58	m	03:38	−	+	−	AVS	HIT normal	None	0	Normal	−	−	n.p	Transient vestibular episode (remission of symptoms < 24 h, vHIT normal)	0	Yes
NoIVT-02	50	f	01:57	−	−	−	AVS	Upbeat nystagmus	Tetraataxia	2	Normal	Occlusion of right VA (hypoplast.)	−	MRI: normal (MRA: distally occluded hypoplast. VA right)	Wernicke encapholpathy (remission of symptoms under substitution of Thiamin B1)	0	Yes
NoIVT-03	77	m	01:36	−	−	−	AVS	HIT normal	Mild dysarthria	1	Normal	−	−	n.p	Vestibular neuritis (vHIT right abnormal)	0	Yes
NoIVT-04	66	m	02:15	−	−	−	AIS	Vertical gaze palsy (upward), HIT normal	None	0	Normal	−	−	MRI: normal	Transient vestibular episode (remission of symptoms < 24 h, vHIT normal)	0	Yes
NoIVT-05	75	f	02:23	−	−	−	AIS	Not reported	Dysarthria, unilateral facial weakness	3	Normal	−	−	MRI: stroke in paramedian pons	Pontine stroke	0	Yes
NoIVT-06	63	m	03:49	−	+	−	AVS	HIT normal	None	0	Normal	Normal	−	n.p	Transient vestibular episode (remission of symptoms < 24 h, vHIT normal)	0	Yes
NoIVT-07	77	f	02:03	+	+	+	AVS	HIT normal	Hemiataxia	1	Normal	−	−	n.p	Brainstem stroke (vHIT normal)	1	Yes
NoIVT-08	50	m	03:10	−	−	−	AVS	Pure torsional nystagmus, bilateral GEN, abducens palsy left, miosis left	Hemihypaesthesia	2	Normal	−	−	MRI: stroke in the right dorsolateral medulla oblongata	Medulla oblongata stroke	1	No
NoIVT-09	64	f	00:59	−	+	−	AVS	HIT normal	None	0	Normal	−	−	n.p	Brainstem stroke	0	Yes
NoIVT-10	51	m	01:03	−	−	−	AIS	Skew deviation	Unilateral facial weakness	1	Normal	Normal	−	MRI: small stroke in Mesencephalon	Stroke in mesencephalon	1	Yes
NoIVT-11	77	f	03:58	−	−	−	AVS	HIT normal	None	0	Normal	−	−	MRI: normal	Transient vestibular episode (remission of symptoms < 24 h)	0	Yes
NoIVT-12	80	m	01:37	+	−	−	AVS	HIT normal	None	0	Normal	Normal	−	MRI: stroke SCA left	Cerebellar stroke (SCA left)	0	Yes
NoIVT-13	52	m	01:19	−	+	−	AIS	HIT normal, bilateral GEN	Hemiataxia	1	Normal	VA occlusion V3/4 left	−	MRI: stroke PICA left	Cerebellar stroke (PICA left)	0	Yes
NoIVT-14	39	m	01:05	+	+	+	AVS	HIT normal, bilateral GEN	None	0	Normal	−	−	MRI: stroke PICA right	Cerebellar stroke (PICA right) with PFO	0	Yes

When comparing the baseline characteristics between the two groups (Table 3), there was no difference between IVT and NoIVT with regards to demographic parameters (age, sex), comorbidities/vascular risk factors, time since symptom onset and the results from the clinical oculomotor examination (apart from a higher percentage of patients with a normal (central) head impulse test in the NoIVT group).

**Table 3:** Baseline characteristics of the IVT and the NoIVT group

Characteristics	IVT group (*n* = 15)	NoIVT group (*n* = 14)	Stat (*p*)
Demographics			
Age [years, mean ± SD]	65 ± 13	63 ± 13	n.s
Sex (female/male)	5/10 (33/67)	5/9 (36/64)	n.s
Comorbidities/vascular risk factors			
Diabetes	2 (13)	2 (14)	n.s
Arterial hypertension	9 (60)	10 (71)	n.s
Prior stroke	1 (7)	2 (14)	n.s
Previous diagnosis of a vestibular disorder	0 (0)	1 (7)	n.s
Time period between onset and ED arrival			
Time since symptom onset [h:min]	1:47	2:12	n.s
Disabling vestibular symptoms			
Perception of severe dizziness/vertigo	12 (80)	3 (21)	**0.002**
Nausea and vomiting	5 (33)	6 (43)	n.s
Inability to stand unsupported	8 (53)	2 (14)	**0.027**
Clinical examination findings			
Spontaneous nystagmus (AVS)	10 (67)	10 (71)	n.s
Central oculomotor signs, any	12 (80)	11 (79)	n.s
HINTS positive/central	7 (47)	11 (79)	n.s
Normal (central) head impulse test	3 (20)	9 (64)	**0.016**
Nystagmus alternating with gaze	2 (13)	3 (21)	n.s
Skew deviation	3 (20)	1 (7)	n.s
Ptosis, Horner sign	3 (20)	0 (0)	n.s
Central pattern of spontaneous nystagmus	3 (20)	1 (7)	n.s
Gaze palsy, ophthalmoparesis	4 (27)	2 (14)	n.s
Focal neurological abnormalities, any^a^	13 (87)	6 (43)	**0.009**
Dysarthria	9 (60)	2 (14)	**0.004**
Hemiataxia	10 (67)	3 (21)	**0.014**
Mild unilateral facial weakness	0 (0)	2 (14)	n.s
NIHSS score at admission (mean ± SD)	1.9 ± 1.2 (range 0–4)	0.8 ± 1.0 (range 0–3)	**0.004**

Bold values indicate statistically significant results of between-group comparisons (*p* < 0.05)

Data are *n* (%) unless otherwise indicated

*AVS* acute vestibular syndrome, *HINTS* signs include head impulse, nystagmus, test of skew deviation, *NIHSS* National Institute of Health Stroke Scale

^a^Due to the predefined exclusion criteria, major deficits such as hemiparesis, aphasia/anarthria or hemianopia were not present in this study cohort

Patients, who were decided to receive IVT, more often had the perception of severe dizziness/vertigo and an inability to stand unsupported than NoIVT patients (please see Table 3 for numbers and statistical comparisons). Furthermore, they more frequently revealed focal abnormalities on the neurological examination than NoIVT patients (87% versus 43%). In line with that, the NIHSS score was higher in the IVT than in the NoIVT group (1.9 versus 0.8).

Regarding the imaging studies performed at admission, both groups did not differ with respect to the number of NCCT studies conducted and their results (especially acute ischemic stroke).

Both CTA and CTP studies were significantly more often conducted in IVT patients than in NoIVT patients (Table 4). CTP studies were performed in 80% of the IVT patients (versus 0% of NoIVT patients). A focal hypoperfusion in the posterior circulation was discovered in 7 of 12 (58%) IVT patients who received CTP. For the CTAs performed, the likelihood of a pathological result (stenosis or occlusion of a vertebral or the basilar artery) was not significantly different between IVT and NoIVT patients.

**Table 4:** Results of imaging studies and clinical outcome of IVT versus NoIVT patients

Parameters	IVT group (*n* = 15)	NoIVT group (*n* = 14)	Stat (*p*)
Results of initial imaging studies (before decision on IVT)			
Non-contrast CT (NCCT) performed	15 (100)	14 (100)	n.s
Acute ischemic lesion on NCCT	1 (7)	0 (0)	n.s
CT angiography (CTA) performed	15 (100)	5 (36)	**< 0.001**
Stenosis/occlusion in the posterior circulation	8 (53)	2 (14)	n.s
CT perfusion (CTP) performed	12 (80)	0 (0)	**< 0.001**
Hypoperfusion in areas of the posterior circulation	7 of 12 (58)	–	–
Results of follow-up imaging studies			
Imaging performed			
Any (CT or MRI)	15 (100)	9 (64)	**0.011**
MRI	6 (40)	9 (64)	n.s
CT	9 (60)	0 (0)	**< 0.001**
Acute stroke lesion confirmed	7 (47)	6 (43)	n.s
Intracerebral hemorrhage (ICH)^a^	0	0	n.s
Follow-up CT result in patients with hypoperfusion on initial CTP (*n* = 7)			
Area of infarction congruent with area of hypoperfusion on CTP	3	–	
Infarction absent or smaller than hypoperfused area on CTP	3	–	
TTD-CBF discrepancy and no infarction	1	–	
Final diagnosis and clinical outcome			
Diagnosis at discharge			
Ischemic stroke	13 (87)	8 (57)	n.s. (0.075)
Cerebellar	4 (27)	3 (21)	n.s
Brainstem (incl. pons, medulla obl.)	7 (47)	4 (29)	n.s
Mesencephalon/thalamus	2 (13)	1 (7)	n.s
Non-stroke diagnosis	2 (13)	6 (43)	n.s. (0.075)
Transient vestibular episode	1 (7)	4 (29)	n.s
Vestibular neuritis	1 (7)	1 (7)	n.s
Wernicke encephalopathy	0 (0)	1 (7)	n.s
NIHSS at discharge (mean ± SD)			
All patients	1.1 ± 1.1	0.2 ± 0.4	**0.010**
Patients with final stroke diagnosis	1.3 ± 1.1	0.4 ± 0.5	**0.044**
NIHSS improvement to admission (mean ± SD)			
All patients	0.8 ± 1.1	0.6 ± 0.9	n.s
Patients with final stroke diagnosis	0.8 ± 1.1	0.6 ± 1.1	n.s
Discharged home, able to walk independently (no in-patient rehabilitation)			
All patients	11/15 (73)	13/14 (93)	n.s
Patients with final diagnosis of stroke	9/13 (69)	7/8 (88)	n.s

Bold values indicate statistically significant results of between-group comparisons (*p* < 0.05)

Data are *n* (%) unless otherwise indicated

^a^Intracerebral hemorrhage according to the SITS-MOST definition of a parenchymal hematoma (PH-2) [40]

### Clinical and imaging outcome in IVT and NoIVT patients

Although absolute numbers differed, the final diagnoses given at discharge were not significantly different between the IVT and NoIVT group (Table 4). In particular, a PCS was diagnosed in 87% of IVT patients versus 57% of NoIVT patients (*p* = 0.075), a non-stroke diagnosis (including vestibular neuritis, Wernicke encephalopathy and transient vestibular episodes) was diagnosed in 13% of IVT and in 43% of NoIVT patients (*p* = 0.075).

Regarding the clinical outcome (Table 4), the mean NIHSS score at discharge was slightly higher in IVT than NoIVT patients, but the improvement in relation to the NIHSS score at admission did not differ between IVT (0.8 ± 1.1) and NoIVT (0.6 ± 0.9). The percentage of patients who could be discharged home, being able to walk independently, was not significantly different between IVT (73%) and NoIVT (93%). The comparisons yielded similar results when the analyses included only those patients who finally received the diagnosis of ischemic stroke at discharge (*n* = 13 of IVT patients, *n* = 8 of NoIVT patients).

One patient in the IVT group died in the hospital due to cardiac arrest several days after IVT administration, hence this death was not associated to IVT treatment.

With respect to imaging outcome parameters, the number of acute stroke lesions confirmed by follow-up imaging did not differ between IVT (47%) and NoIVT (43%). There was no (symptomatic) ICH occurring in either group. There was only one patient in the IVT group who revealed a slight hemorrhagic transformation of the infarcted brain tissue but this did not fulfill the SITS-MOST criteria of a symptomatic intracranial hemorrhage [40].

**Fig. 1:**
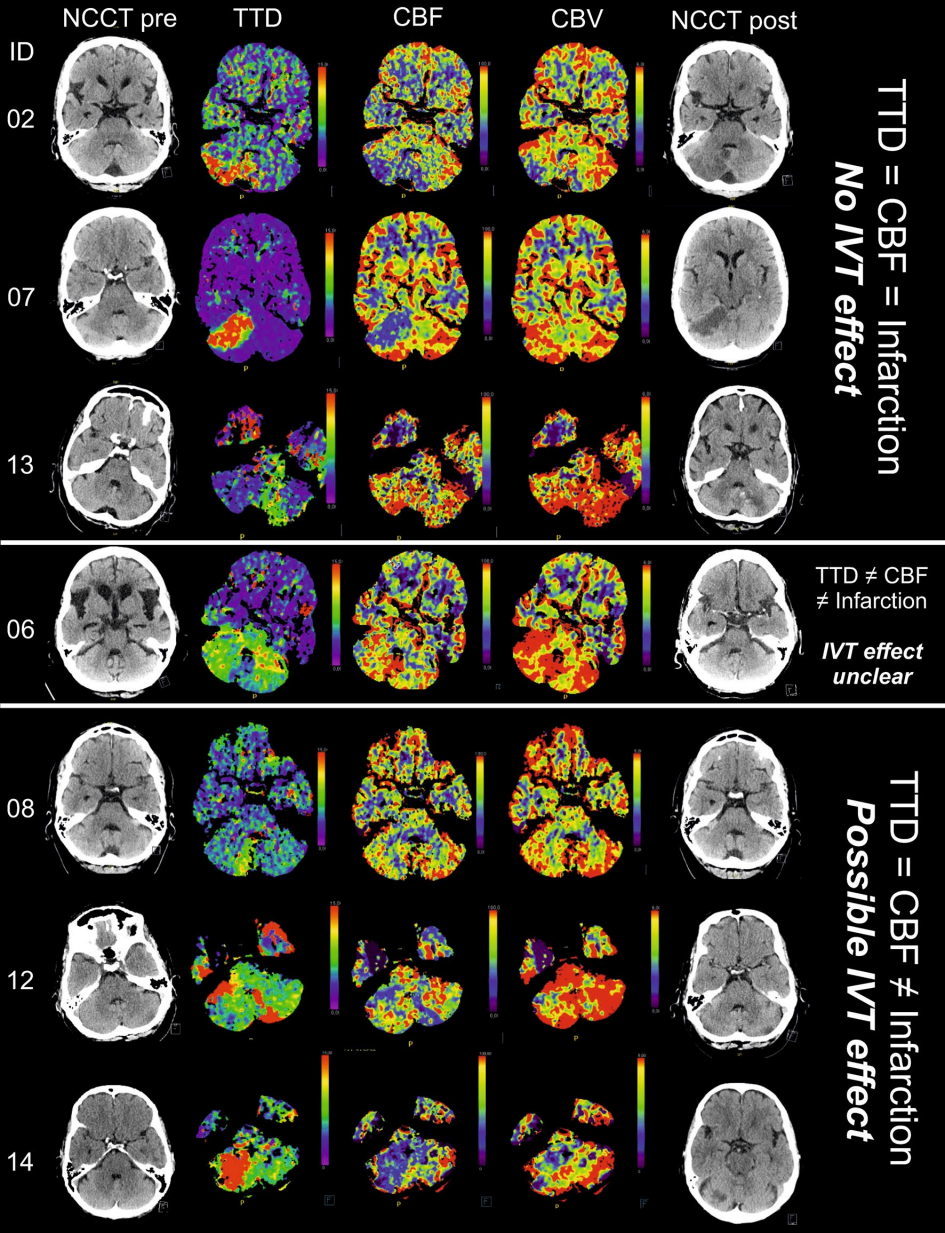
The effect of intravenous thrombolysis (IVT) with regards to imaging parameters: non-contrast CT (NCCT) and CT perfusion (CTP) as well as follow-up NCCT for IVT-treated patients with CTP abnormalities. Despite IVT treatment, three patients (ID 02, 07, 13) finally revealed stroke lesions on follow-up NCCT that matched the areas of hypoperfusion on initial CTP [prolongation of the time-to-drain (TTD) and reduction of cerebral blood flow (CBF)]. Hence, IVT seemed to have no effect in these patients, at least with regards to CT imaging parameters. One patient (ID 06) initially revealed a TTD prolongation but normal CBF in areas of the posterior circulation and there was no infarction on follow-up NCCT. The IVT effect here is unclear. The remaining three patients (ID 08, 12, 14) may have benefited from IVT with regards to imaging parameters. There was either no stroke lesion on follow-up NCCT (ID 08, 12) or an area of infarction that was smaller (ID 14) than the initial CTP abnormality would have predicted

For those IVT-treated patients who had received CTP at admission (*n* = 7), we first compared the area of the initial hypoperfusion (indicated by TTD prolongation) with the final stroke lesion on the follow-up NCCT (Fig. 1). Three out of seven patients (# 02, 07, 13) had an area of infarction on follow-up CT that congruently matched the hypoperfused area on the initial CTP. Two patients (# 08, 12) had no and one patient (# 14) a clearly smaller stroke lesion on the follow-up CT than one would have expected from the initial perfusion deficit. One IVT patient (# 06) exhibited an unspecific finding with prolonged TTD and discrepantly normal CBF on the initial CTP and no infarction on follow-up CT.

When comparing the individual CBF and CBV maps (Fig. 1), four patients in the IVT group (# 07, 08, 12, 13) revealed a mismatch between a focally reduced CBF versus a normal CBV. Two of these patients (# 07, 13) later revealed an infarction on follow-up NCCT at the site of the initial CBF reduction, whereas the other two patients (# 08, 12) revealed no stroke lesion on follow-up NCCT. One patient (# 14) initially showed a circumscribed area of CBV reduction that was smaller than the area of reduced CBF, on the follow-up NCCT, there was a stroke lesion that matched the small area of CBV reduction. One patient (# 02) showed a congruent area of focal CBF and CBV reduction that fitted the final stroke lesion on follow-up NCCT. One patient (# 06) showed an area of isolated TTD prolongation but no pathology on CBF and CBV maps and no infarction on follow-up NCCT.

## Discussion

In our retrospective single-center investigation of patients who presented to the ED with dizziness as the main complaint, only about 4% of patients had an AVS/AIS with (mild) clinical signs indicative of PCS and were otherwise eligible for IVT (symptom onset < 4.5 h ago, no ICH on NCCT). That there was such a relatively small number of IVT-eligible patients in the cohort is well in line with previous findings that (1) only about 11% of patients presenting with an AVS/AIS to the ED have an ischemic stroke [14] and that PCS patients, commonly presenting with acute dizziness, arrive later at the hospital than ACS patients and more often exceed the time window of 4.5 h for IVT treatment [29].

About half of them received IVT (IVT group, *n* = 15), while the others were admitted to the stroke unit without IVT treatment (NoIVT group, *n* = 14). Due to our exclusion criteria, none of the AVS/AIS patients treated with IVT had a major neurological deficit (aphasia, hemiparesis, or hemianopia) that per se would have indicated IVT. Since an isolated vestibular syndrome or unsteadiness of stance/gait is often not regarded as disabling enough to justify IVT [18, 27], we specifically questioned whether the clinician’s decision on IVT in these patients was influenced by additional factors, e.g., individual vascular risk profile, (mild) focal neurological deficits or particular imaging abnormalities that may have pointed to brain tissue at risk. Furthermore, we were interested in between-group differences concerning the short-term clinical outcome, final diagnosis at discharge (stroke versus non-stroke), and symptomatic ICHs as well as the question whether IVT may have led to a potential salvage of brain tissue at risk.

### Which clinical or imaging parameters trigger IVT in AVS/AIS patients with suspected PCS?

Patients who received IVT did not differ from NoIVT patients with respect to demographic features (age, sex), vascular risk factors (arterial hypertension, diabetes, and prior stroke), time between symptom onset and ED presentation. Hence, the individual past medical history, comorbidities, or vascular risk profile seem not to have influenced the decision-making on IVT. In contrast, disabling vestibular symptoms (i.e., the perception of severe dizziness/vertigo and the inability to stand unsupported) were more often present in IVT patients than in NoIVT patients. Hence, it could be assumed that the presence of severe and disabling vestibular symptoms contributed to the clinician’s decision in favor of IVT. Furthermore, IVT patients more often revealed (mild) focal neurological abnormalities (e.g., dysarthria, hemiataxia) leading to slightly higher NIHSS scores at admission, compared to NoIVT patients. Hence, the presence of (mild) focal neurological deficits and the consecutively increased NIHSS score in AVS/AIS patients appeared to have directed (or at least contributed to) the decision pro IVT. However, it remains open whether these signs just convinced the clinician of a central etiology of the AVS/AIS (as opposed to a peripheral vestibulopathy) or whether they were regarded as symptoms disabling enough to justify IVT treatment.

With respect to brain imaging studies performed at admission, IVT differed from NoIVT patients in that they more frequently received CTA (100% versus 36%) and CTP (80% versus 0%) imaging. This may simply reflect the fact that an acute and disabling PCS was more often clinically suspected in IVT patients than in NoIVT patients and that CT angiography and CT perfusion imaging was more often asked for in these patients to urgently assess the posterior circulation. On the other hand, it may also have been the result of the additional imaging study that may have been an important trigger in favor of IVT treatment. Notably, CTP revealed hypoperfusions in the VBS territory in seven out of twelve patients who received IVT. Due to the retrospective design of our study, we cannot rule out that these seven patients may also have been treated with IVT without showing a critical ischemia on CTP. However, the CTP hypoperfusion may also have been taken as the final argument that the vestibular syndrome was really due to an acute PCS with brain tissue at risk (and not peripheral vestibulopathy) which may have eventually encouraged IVT administration. Notably, a CBF–CBV mismatch on initial CTP seemed not to be a crucial factor for indicating (or withholding) IVT and had no clear prognostic impact for the final stroke lesion, at least not in the small cohort of this study. Only four of the seven patients treated with IVT revealed a CBF–CBV mismatch, half of them showing a stroke lesion on follow-up NCCT that matched the (larger) area of the initial CBF reduction.

Current guidelines state that non-contrast CT (NCCT) is sufficient to decide on the use of IVT in otherwise eligible patients with suspected acute ischemic stroke within the 4.5 h time window [27]. However, focal hypoperfusion on the initial CTP in PCS patients can predict larger final infarct volume on follow-up imaging [10] as well as worse 12-month clinical outcome [25]. Furthermore, concerning the guidance of therapy, CTP was previously shown to allow extending the time window of IVT (up to 9 h after symptom onset) in CTP-selected patients with an acute ischemic stroke of significant clinical severity (score of 4–26 on the NIHSS) [20].

Taken together, the data suggest that CTP can support decision-making on IVT in less severe PCS presenting as AVS/AIS. However, further evidence must be obtained in larger prospective studies. Our study cannot answer the question, whether the CBF–CBV mismatch concept, which is used for identifying tissue at risk and indicating IVT in extended or unclear time windows in ACS patients [20], could also be helpful in PCS patients.

### How frequent are IVT-related intracerebral hemorrhages and final non-stroke diagnoses in AVS/AIS patients with suspected PCS?

In our study cohort of 29 patients with AVS/AIS and suspected PCS in the ED, eight (28%) patients eventually received a non-stroke diagnosis at discharge, including vestibular neuritis, Wernicke encephalopathy, and transient vestibular episode. In the group of 15 IVT-treated patients, 1 patient had a vestibular neuritis and one patient a transient vestibular episode that remitted within 1.5 h after admission. The entity of a transient vestibular episode, however, is hard to disentangle from a transient ischemic attack (or even an acute ischemic stroke that was successfully treated by early IVT application), because diagnosis is mainly based on the patient’s history and first clinical assessment in the ED, while the MRI and quantitative head impulse testing are usually normal. From a previous study using early MR perfusion imaging, we know that 27% of patients with an acute transient vestibular syndrome actually have an ischemic vascular etiology [5].

When excluding transient vestibular episodes, only 1 (7%) of 15 patients treated with IVT finally received a non-stroke diagnosis which is in the range of previously reported percentages of stroke mimics (1.4–14%) in IVT-treated patients with suspected ischemic stroke [11, 41].

Concerning safety aspects, in our cohort of 15 IVT-treated AVS/AIS patients, not a single one developed a symptomatic ICH. This is in line with previous reports on an overall reduced likelihood of symptomatic ICHs in PCS as compared to ACS [31].

### Do PCS patients with AVS/AIS benefit from IVT?

Due to the retrospective design and the acute phase setting of our study, we did not have access to long-term (e.g., 90-day or 1-year) outcome measures in our patients. Instead, we analyzed the short-term outcome including the percentage of patients who finally could be discharged home, able to walk independently and without the need for further in-patient rehabilitation. We also analyzed the change (improvement) of the NIHSS score from admission to discharge. Regarding both parameters, patients in the IVT group did not differ significantly from patients in the NoIVT group. Taken together, our data on the clinical outcome in a relatively small cohort do not indicate that patients with an AVS/AIS and suspected PCS benefit from IVT (nor are they harmed).

With regards to imaging parameters, three of seven IVT patients revealed a discrepancy between the critically ischemic brain area (hypoperfusion) revealed on the initial CTP and the final stroke lesion on follow-up NCCT, which was either smaller or completely absent. These PCS patients may have benefited from early thrombolysis with regard to a potential salvage of brain tissue at risk. The significance of this result is certainly limited because of the small number of patients and because CT imaging is less sensitive than MRI for revealing the total extent of damaged brain tissue, especially for strokes in the posterior circulation [3, 28]. However, IVT-related salvage of brain tissue in PCS was also previously shown by use of MR perfusion imaging in two cases of cerebellar strokes who finally revealed a smaller stroke lesion than it was indicated by the initial area of hypoperfusion [16].

### Should AVS/AIS patients be thrombolysed if PCS is suspected clinically or by use of CT perfusion imaging?

The benefit of IVT within 4.5 h after symptom onset is well established for adult patients with *disabling* stroke symptoms regardless of patient’s age and stroke severity [19, 30]. Thus, according to current guidelines, IVT is also recommended in otherwise eligible patients with only mild but disabling stroke symptoms [27]. However, IVT is not recommended in patients with mild (NIHSS 0–5) but non-disabling stroke symptoms, as the potential risk then exceeds the anticipated benefit [27].

For acute vestibular patients, Lee and Kim proposed that “isolated vascular vertigo is not indicated for thrombolysis due to its low disability score” and that “patients with AVS due to stroke should [only] be considered for intravenous thrombolysis or acute endovascular surgery when the NIHSS is > 4 or in cases with lower NIHSS that will clearly produce significant disability”, whereas “conservative treatments […] may be sufficient for AVS in isolation or AVS associated with minimal disability such as internuclear ophthalmoplegia” [18]. However, the NIHSS is known to underestimate disability in PCS patients [2, 37], and those presenting with a severe unsteadiness (or even inability) of stance and/or gait and maybe unilateral limb ataxia would still score less than 4 points on the NIHSS. This leads to the critical question whether a PCS presenting with an isolated AVS/AIS and only central oculomotor signs but no major focal neurological abnormalities produces “significant disability”. It should be considered that not only postural stability, spatial orientation, navigation and visual exploration in everyday life might be impaired with oculomotor abnormalities but also that the imposed restrictions (e.g., of the patients’ driving license) might lead to social and functional disability.

However, from longitudinal studies in patients with isolated cerebellar strokes, we know that these patients generally have a favorable clinical outcome without acute IVT treatment [24, 26]. This could be an argument to withhold IVT in the acute stage of less severe PCS strokes, because the potential clinical benefit in the long-term is too small that it may not weigh up the risk of IVT-related harm (e.g., ICH) in the acute stage. This is in line with the data of our retrospective study, where it appeared that IVT was rather administered in patients with additional focal neurological abnormalities and only rarely in patients with an isolated AVS/AIS despite the assumption of an underlying PCS. The question, however, whether or which AVS/AIS patients with suspected PCS should be thrombolysed or not, can only be answered in controlled clinical trials.

### Limitations

Due to the retrospective character and real-world setting in which the data for this study were acquired [21], there are implicit limitations. First, the use of imaging methods (NCCT, CTP, and MRI) did not follow a uniform protocol but depended on the individual decision of the responsible clinician. Particularly, a hypoperfusion could only be detected in patients who received CTP imaging and NCCT, if performed as the sole imaging method in a patient, was certainly inferior to MRI in assessing the presence and size of an ischemic stroke lesion [3]. However, the diagnosis (stroke or non-stroke) at discharge was clinically made and, therefore, not (exclusively) based on the brain imaging results. Hence, while an individual clinical misdiagnosis cannot be completely ruled out, differences between subgroups concerning the use and modality of brain imaging studies or the potential prevalence of “MRI-negative” ischemic strokes [8] could not crucially affect our findings and conclusions.

## Conclusion

Among the acutely dizzy patients presenting to the ED, only few have PCS and are eligible for IVT. Decision-making on IVT in AVS/AIS patients with suspected PCS appears to be influenced by the presence of severe and disabling vestibular symptoms and additional focal abnormalities revealed on the neurological examination. Moreover, perfusion imaging seems to assist in making the PCS diagnosis in AVS/AIS patients and may help to identify brain tissue at risk. In our relatively small sample, there was no evidence for a significant clinical benefit or harm (ICH) of IVT treatment in PCS patients presenting with AVS/AIS. However, future studies are needed to prospectively investigate the question whether IVT is effective and safe in PCS patients presenting with AVS/AIS and whether perfusion imaging is a reliable tool in the stratification for IVT treatment.
